# Digital Technology: Opportunity or Challenge?

**DOI:** 10.4269/ajtmh.23-0804

**Published:** 2024-01-16

**Authors:** Kaiwen Zhang, Hanruo Liu

**Affiliations:** Beijing Institute of Ophthalmology Beijing Tongren Eye Center Beijing Tongren Hospital Capital Medical University Beijing, China E-mail: 18811155900@163.com; Beijing Institute of Ophthalmology Beijing Tongren Eye Center Beijing Tongren Hospital Capital Medical University Beijing, China; School of Medical Technology Beijing Institute of Technology Beijing, China; National Institutes of Health Data Science at Peking University Beijing, China E-mail: hanruo.liu@hotmail.co.uk

Dear Sir,

We read with great interest the paper by Varo and colleagues, which applied 3D printing technology to design a smartphone-based retinal imaging device and presented a population-based pilot study in Mozambique.^1^ The study demonstrated the potential role of smartphones and telemedicine for screening and diagnosis of fundus lesions in areas where eye health care resources are scarce.

Accessibility and equity of eye care services remains a major challenge for global eye health, and the development of digital technologies such as telemedicine provides an effective solution.^2^ In low- and middle-income countries, telemedicine-based ophthalmic screening will help to alleviate the unequal distribution of healthcare resources between regions, working toward full coverage of high-quality eye health. As shown in the study by Varo et al, with the technical support of telemedicine and mobile phones, there is the potential to implement large-scale, population-based retinopathy screening in remote areas, obtaining epidemiological data that could guide the development of health policies and clinical treatment protocols.^1^ Compared with manual screening, remote screening has unique advantages such as low cost, convenience, and high efficiency, but its wide-scale promotion at the grass-roots level has the following difficulties that require our attention.

First, the impact of novel digital technologies on the environment should be viewed using “systems thinking.” Telemedicine is known to be effective in reducing travel-related carbon emissions. However, it is worth noting that information and communication technology accounts for 3.7% of global carbon emissions.^3^ It is often customary to ignore the environmental impacts generated by innovative technologies, An increase in the demand for digital healthcare will inevitably lead to a significant increase in digital technology-related carbon emissions. We therefore call for the promotion of digital health technologies to be accompanied by harmonized standards for quantifying and tracking their carbon emissions, to promote the widespread adoption of environmentally sustainable digital health solutions.

Second, equity in digital healthcare should be based on the underlying core design. On the one hand, the promotion of digital screening may lead to a Matthew effect, whereby those who are advantaged gain increasing advantage over time, due to the existence of an access and utilization divide. Given limited public health funding, we suggest promoting the acceleration of digital ophthalmology technology at the grassroots level through non-governmental organizations to ensure the inclusiveness of digital healthcare. On the other hand, as mentioned by the authors, the development and application of digital medical devices needs to be based on population-wide pilots, with improved compatibility through optimization of the devices.

Third, the digital age without walls cannot be without borders. With the development of cloud platforms, 5G communication technologies, and lifelong electronic health records, information storage and transmission have become more convenient, yet users are becoming concerned about potential privacy issues.^4^ While promoting the construction of digital healthcare, it is important to set up appropriate regulatory agencies and develop strict privacy policies. Transparency in the entire process of information flow should be achieved to dispel the public’s privacy concerns and establish solid trust to promote the wide-scale application of digital technology and maximize the advantages of digital healthcare.

Furthermore, the scaling up of innovative digital interventions needs to be supported by economic evidence. Comparisons of the performance and efficiency of different screening devices should be accompanied by comprehensive consideration of their economic benefits.^5^ Given the limited resources and financing for ophthalmic care, there is a need for real world-based economics assessment of digital medical technologies to develop cost-effective intervention programs according to the level of economic development of different regions and the disease characteristics of different populations, and to provide support for rational allocation of health resources.

In conclusion, we believe that digital technology will enable low-cost population-wide eye disease screening and facilitate the achievement of universal eye health coverage. However, further research is needed to ensure the accessibility, privacy, affordability, and sustainability of digital ophthalmology.
